# Driver Identification Using Statistical Features of Motor Activity and Genetic Algorithms

**DOI:** 10.3390/s23020784

**Published:** 2023-01-10

**Authors:** Carlos H. Espino-Salinas, Huizilopoztli Luna-García, José M. Celaya-Padilla, Jorge A. Morgan-Benita, Cesar Vera-Vasquez, Wilson J. Sarmiento, Carlos E. Galván-Tejada, Jorge I. Galván-Tejada, Hamurabi Gamboa-Rosales, Klinge Orlando Villalba-Condori

**Affiliations:** 1 Unidad Académica de Ingeniería Eléctrica, Universidad Autónoma de Zacatecas, Jardín Juarez 147, Centro, Zacatecas 98000, Mexico; 2 CONACYT, Universidad Autónoma de Zacatecas, Jardín Juarez 147, Centro, Zacatecas 98000, Mexico; 3 Ingeniería Mecanica, Universidad Continental, Arequipa 04002, Peru; 4 Ingeniería en Multimedia, Universidad Militar de Nueva Granada, Cra 11, Bogotá 101-80, Colombia; 5 Vicerrectorado de Investigación, Universidad Católica de Santa María, Arequipa 04002, Peru

**Keywords:** driver identification, genetic algorithms, feature extraction, ADAS, random forest

## Abstract

Driver identification refers to the process whose primary purpose is identifying the person behind the steering wheel using collected information about the driver him/herself. The constant monitoring of drivers through sensors generates great benefits in advanced driver assistance systems (ADAS), to learn more about the behavior of road users. Currently, there are many research works that address the subject in search of creating intelligent models that help to identify vehicle users in an efficient and objective way. However, the different methodologies proposed to create these models are based on data generated from sensors that include different vehicle brands on routes established in real environments, which, although they provide very important information for different purposes, in the case of driver identification, there may be a certain degree of bias due to the different situations in which the route environment may change. The proposed method seeks to intelligently and objectively select the most outstanding statistical features from motor activity generated in the main elements of the vehicle with genetic algorithms for driver identification, this process being newer than those established by the state-of-the-art. The results obtained from the proposal were an accuracy of 90.74% to identify two drivers and 62% for four, using a Random Forest Classifier (RFC). With this, it can be concluded that a comprehensive selection of features can greatly optimize the identification of drivers.

## 1. Introduction

Currently, there are systems capable of improving the quality and experience of vehicle users; an example are the Advanced Driver-Assistance Systems (ADAS). These systems have gained considerable attention in the automotive field as enablers for vehicle energy consumption, safety, and comfort enhancement [1]. These systems exploit a large number of sensors available in some vehicles today and serve the driver, alerting him in cases of potential problems. However, their generated data can also be useful for ensuring vehicle safety by recognizing the driver’s identity. This is advantageous for ADAS because it guarantees vehicle security by warning the owner in the event of an unauthorized driver or even theft, as in recent years, vehicle theft has increased around the world. According to the FBI, vehicle theft in 2017 is estimated at 773,139 in the US, a 10.4% increase compared to the 2013 report, which gives a general overview of this problem. In the case of the ADAS systems, it also builds a driver profile table that can customize the sort of assistance to the identity of the driver [2].

There are internal devices that can access the internal computer network of a vehicle to access its data for different purposes. Vehicle-based performance technologies infer driver behavior by constantly monitoring some elements of the car, such as lane deviation, steering, or speed variation; such systems are critical for detecting and preventing drowsiness [3]. Drowsy driving is one of the main causes of traffic accidents; since drivers cannot react to dangerous situations when drowsy, major accidents can occur. To prevent accidents due to drowsy driving, it is necessary to detect driver drowsiness early [4]. Another important case where the monitoring of driver behavior through vehicle sensors can help improve driver safety is the detection of erratic driving, as recently the exposure to different factors that influence this type of driving has increased, some of these factors may be the distraction by cell phone, alcohol, and drug use, to name few [5].

It is necessary to go through the different applications and implications involved in the analysis and processing of motor activity or diver behavior data, in order to create driver identification systems that are less invasive and less susceptible to failures derived from uncontrolled conditions, taking advantage of the features provided by some sensors such as vehicle speed, Revolutions Per Minute (RMP) engine load, and even steering wheel variation, where is it used driving behavior analysis [6]. Moreover, Global Positioning System (GPS) is commonly deployed in car navigation systems and can also be used to learn an individual’s driving pattern, which makes it applicable in real-time applications with the least overhead costs for different resources [7], and it provides accurate position and velocity information when there is a direct line of sight to four or more satellites [8]. However, the satellite signal can be blocked in dense urban scenarios such as urban canyons and tunnels [9]. This is a major problem because GPS may suffer from interruptions and outages. On the other hand, there are biometric systems that use the information generated through the body for driver identification, a technology that records individual physical and behavioral features, and recognizes them in real-time [10]. Therefore, several identification systems are being researched that use biosignals such as Electrocardiogram (ECG); however, the ECG signal acquired in the driving environment contains artifact noise from some movement depending on the complex state of the driver [11].

Driving patterns simulated as those of a real environment have been studied in the state-of-the-art using different features extracted mainly from the vehicle’s Controller Area Network (CAN-bus), which facilitates communication between the electronic control units placed in the car. It is one of the gadgets that offer crucial information on the internal behavior of a vehicle. The main features extracted from CAN-bus are the steering wheel, the vehicle speed, and the engine speed, etc. These features may range from one to twelve. The use of CAN-bus technology, which provides access to data on the order of several thousand signals that capture details about the car and its surroundings at a frequency in the sub-Hertz range, has become a standard for embedded systems in automobiles [12]. Such applications gain from capturing driving style from behind-the-wheel data without raising costs or infringing driver privacy [13]. Moreover, in-vehicle CAN-bus data has been found to be significantly accurate and reliable for driver profiling [12]; furthermore, it has been analyzed and validated for driver identification and tracking [14].

Currently, there are few research works that seek to evaluate intelligent feature selection methods based on the importance of the driver identification task using motor activity data related to the main elements of vehicles to optimize the training of machine learning algorithms for classifications of different drivers. There are several feature selection methods that seek to optimize complex problems involving a large amount of information, reducing their dimensionality. Genetic algorithms (GA) have proven to be a tool that produces better classification confidence to solve problems such as driver distraction [15], identification of significant factors in fatal-injury highway crashes [16], optimization of fuel [17], and speed bump detection [18]. In spite of this, the implementation of these algorithms in driver identification has not been explored to date according to the literature review. Therefore, we propose a novel methodology for objective driver identification with an intelligent feature selection in simulated environments with GA, avoiding noise in the data generated by different situations that can occur in real environments. Our approach is based on multivariate genetic algorithms fed with information from a statistical feature of the steering wheel angle and the amount of pressure exerted on the acceleration and braking pedals obtained from the driving simulator; this is in order to obtain the most important features instead of entering all the features to the machine learning algorithms, thus having a novel method that is simple to implement and robust driver identification models for different types of drivers and vehicles.

The rest of the paper is organized as follows: Section 2 explains the materials and methods used to obtain the feature extraction, the genetics algorithms implemented, and the validations’ metrics. Section 3 presents the results obtained from the proposed methodology. Section 4. the results obtained in the previous section are discussed in order to highlight the contribution of the research in relation to the state of the art. Finally, Section 5 concludes and proposes future work for improvement of identification systems.

### Related Work

The related work to driver identification where different methodologies and data capture devices are proposed are mentioned below.

Marchegiani et al. [19] presented a system that uses binary Support Vector Machines operating based on data from the accelerator and brake pedals gathered across several driving sessions as one complete circuit of the track. The CAN-bus messages comprising data on the use of the brake and accelerator pedal signals are processed and arranged into frames for each of these driving sessions, obtaining a recognition rate of over 95%. Jeong et al. [20] proposed a driver identification system using deep learning with CAN-bus data obtained from the vehicle; the proposed system achieves an average accuracy of 90% in an experiment with four drivers. In addition, Ullah et al. [21] uses the driver’s behavior data (with CAN-bus) with the objective to identify the driver of a given unseen driving data, using a lightweight deep learning model obtaining accuracy greater than 90%. Likewise, Ravi et al. [22] proposed optimized deep learning using long and short memory for better training on various data collected from CAN-bus, and the evaluation metrics were calculated on the test data. The performance of the proposed model was compared with a few baseline machine learning models, obtaining an accuracy of 99%, with the proposed model having the best results. Another work that also addressed the same data collection system is that of Hu et al. [23]. They present that, using information from the CAN-bus, a one-dimensional convolutional neural network will identify the drivers. The writers employed the Macro $F_{1}$ score as the criterion for evaluation, and the proposed model’s identification rate among 20 subjects was 99.10%. Nasr et al. [24] presented a methodology to identify drivers based on their behavior analysis. The results with 95% of accuracy demonstrate a competitive performance using CAN-bus data and deep learning. Equally, there are a variety of approaches that use vehicle telematics data [25,26] to improve vehicle safety systems [27,28].

Different measurement and control sensors are installed in vehicles, and the electronic control unit is in charge of them (ECU). The duty of the onboard diagnostic network II (ODB-II) is to transmit data about the vehicle’s primary systems or problem data gathered by the ECU from the onboard sensors [29]. Bernardi et al. [30] considered features from ODB-II available in tested cars to identify drivers. The proposed method is successful in achieving an identification rate of 81% for 12 drivers and 73% for 30 drivers by utilizing a time series classification methodology and a multi-layer perceptron (MLP) network. In addition, Mekki et al. [31] introduced a driver identification model based on collected data from intelligent mobile phones and/or ODB-II using convolutional neural network and recurrent neural network; the model presented an accuracy of 95.1% in the case of 0 anomalies. In the work of Xun et al. [32], to get complete real-time data from the OBD-II port, they used the broadcast feature of the CAN system and an automotive diagnostic tool. The data were then preprocessed using principal component analysis and feature scaling methods. Finally, K-Nearest Neighbor (KNN) and Naive Bayes (NB) algorithms were used for driver identification. The testing findings indicated that all ten drivers may be recognized.

GPS technology has been successfully used by researchers to identify drivers who are using vehicles. GPS signals are available most of the time and are one of the most widely used technologies [33]. Chowdhury et al. [34] investigated if driver identification was possible by using only GPS data measurements. The analysis presented shows that, for the driver identification problem, an average accuracy of 82.3% is achieved. Another work presented by Marchegiani et al. [19] evaluated 15 h of driving data for a total of 416 km traveled, comprising messages from the CAN-bus and GPS traces from different drivers traveling on the same route, obtaining an accuracy of over 95% in rate.

One less explored scenario to date, where conditions can be controlled to obtain more objective results, is driver identification in simulated environments, as presented by Yang et al. [22]. They developed driver identification, using deep learning architecture (Driver2vec), which transfers a brief interval of driving data into an embedding space that represents driver behavior. The information used was gathered from a top-of-the-line driving simulator made by Nervtech. The average time spent in the urban simulator by each driver was 15 min, adding up to a total of more than 15 h of driving. With only a 10-second window of sensor data, the model was able to successfully identify the driver, with an average paired driver recognition accuracy of 83.1%. The potential benefits of incorporating Driver2vec for numerous downstream applications are thus strongly suggested. Meanwhile, Heidecker et al. [35] presented an approach to driver identification in a simulated environment that is useful for many automotive applications. The method is based on the driver’s physiological state (electrocardiogram data). First, they extracted features and trained the Gaussian Mixture Model (GMM) to exploit localities present in the recorded sensor data. Then, they smoothed the noisy signal and reduced the dimensionality for further processing in a one-class Support Vector Machine (SVM) classification. The results obtained on testing data when training the model with one, two, or three drivers were 56.8%, 43.5%, and 37.8% for unknown, driver 1, unknown, driver 1/2, and unknown, driver 1/2/3, respectively.

Although the works related to driver identification in simulated environments are few, the one related to an intelligent selection of features is even less, since in most cases this selection is carried out in a judicious and not objective manner, but there are works in the automotive field where GA is implemented, as is the case of Celay et al. [18]. They developed a novel method for the detection of road abnormalities (i.e., speed bumps). This method makes use of a gyro, an accelerometer, and a GPS sensor mounted in a car. After having the vehicle cruise through several streets, data were retrieved from sensors. Then, using a cross-validation strategy, a genetic algorithm was used to find a logistic model that accurately detects road abnormalities, obtaining an accuracy of 97.14%. Another case of success is that of Song et al. [36], who introduced a learning vector quantization neural network to design the driving patterns recognizer according to a vehicle’s driving information. This multi-mode strategy can automatically switch to the genetic-algorithm-optimized thermostat strategy under specific driving conditions in light and the differences in condition recognition results in the objective to manage the energy of electric vehicles. Moreover, Eraqi et al. [15] presented a publicly available dataset for driver distraction identification and proposed a reliable deep learning-based solution that achieves 91% accuracy. The system consists of a genetically weighted ensemble of convolutional neural networks; they show that a weighted ensemble of classifiers using a GA yields better classification confidence. Finally, Li et al. [16] noted that a fatal-injury crash is a comprehensive result influenced by multiple variables involved at the moment of the crash scenario; the main idea of their paper was to explore the process of significant factors identification. They proposed a data-driven model which combines the non-dominated sorting genetic algorithm with artificial neural network architecture to efficiently search for optimal solutions.

## 2. Materials and Methods

**Figure 1:**
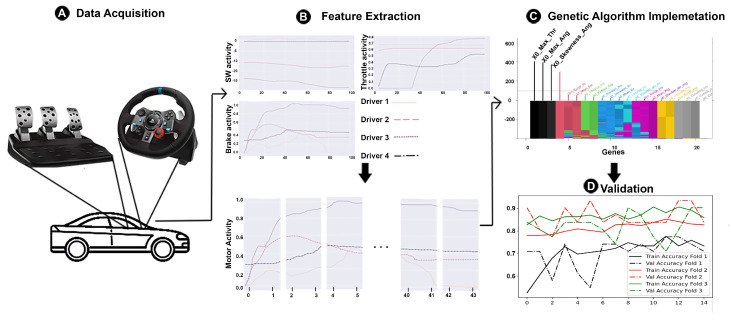
Proposed methodology for the objective identification of drivers by their motor activity based on a genetic algorithm approach.

The following is a brief description of the proposed methodology to be followed for driver identification in a simulated environment. As can be seen in Figure 1, first, in Figure 1A, information on the main elements that allow driving a vehicle are gathered through a CARLA 0.9.13 simulator and devices such as the Logitech G29. In Figure 1B, once the data of interest are obtained, it will be subjected to a data window generation process to extract various statistical features for analysis and processing. Finally, in Figure 1C, an artificial intelligence approach based on GA and Machine Learning (ML) techniques such as Random Forest Classifier (RFC) are implemented to find an efficient model, which can improve the time, computational cost, and accuracy to identify drivers. Each stage is explained in detail in the next subsections.

### 2.1. Data Acquisition

This subsection details the hardware and software used to carry out this work, in addition to justifying the use of simulation environments for the acquisition of data for vehicle user identification.

First, it is necessary to establish a very important aspect regarding data collection, whereby, in general, even if the same route is followed there will be (at least) differences, for example, in traffic conditions, heterogeneity, weather conditions, or elements not foreseen in a test drive in real environments. For this reason, it is proposed to use the CARLA 0.9.13 simulator, which, among its many features, allows controlling in every way the conditions in which driven tests were performed, avoiding unexpected situations and generating low noise and reliable data for analysis. The CARLA simulator is an open-source simulator developed to aid the creation, training, and validation of autonomous vehicles. CARLA attempts to meet the requirements of several ADAS use cases, i.e., training perception algorithms or learning driving policies. CARLA is also free to use and sensor suite configurations that provide signals that can be used to train ML models [37].

On the hardware side, the specifications of the central processing unit consist of a processor Intel Core i5-9400F at 2.90 GHz, 32 GB of RAM, a graphics card NVIDIA GeForce GTX 1070 Ti, and peripherals such as Logitech G29 driving for driving games and driving simulation.

**Figure 2:**
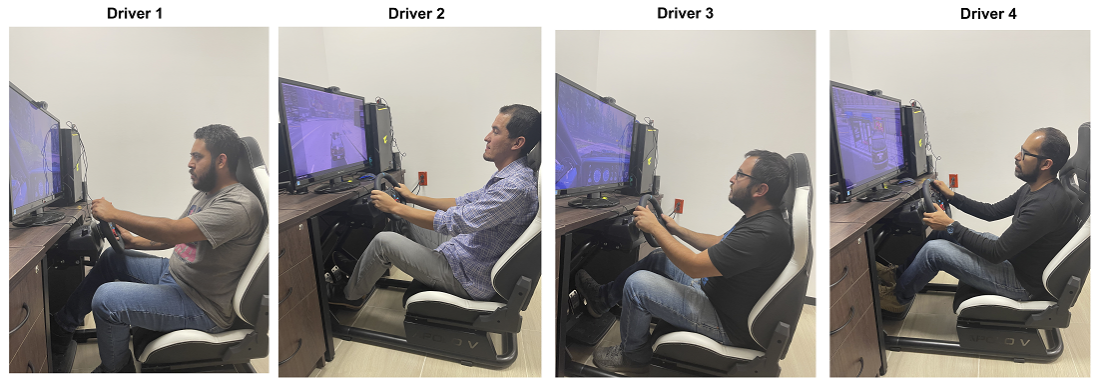
Study subjects performing a driving test in the laboratory of Universidad Autónoma de Zacatecas.

Four study subjects were chosen for data acquisition, who were students from Universidad Autónoma de Zacatecas, aged between 27 and 37 years with an approximate average driving experience of 8.75 years, male, and volunteered to participate in the experiment as shown in Figure 2, having sufficient explanation that includes: the purpose and procedure of the experimentation and the use that was made of the data obtained.

**Figure 3:**
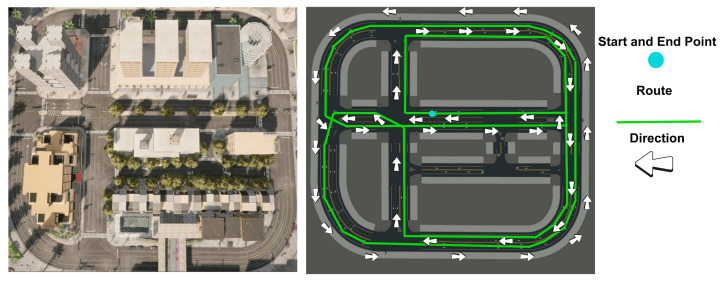
Map of the CARLA simulator used for driving simulation and the established route.

Second, a defined route was established on one of the maps provided by the simulator, as shown in Figure 3, which each of the participants had to follow, respecting all the traffic guidelines that exist in a real environment. It was taken into account to avoid any type of inconvenience, such as the interference of a non-playable character (NPC), which would hinder or cause significant variation in the data.

Even though there are also elements such as traffic lights that were not controlled so that each driver stopped at the same signal at the same time, it was considered to collect only active driving data. According to the work of Ezzini et al. [3], it is possible to identify drivers with very high accuracy within the first 3 min of driving, using a limited amount of sensor data collected from a restricted but judiciously chosen set of the sensor. Despite the experimentation, where conditions are not the same, it gives us a starting point to choose a time interval of 3 min for this research.

Finally, signals extracted from the simulator are those generated through Motor Activity (MA) exercised employing the accelerator pedal, brake pedal, and Steering Wheel Angle (SWA), obtaining 3 variables and 9000 samples (3 min of active driving) per drive as shown in Table 1, where each driver was labeled with 0, 1, 2, 3 for drivers one, two, three, and four, respectively.

### 2.2. Feature Extraction

Most current approaches for driver identification are based on data generated from signals generated by vehicle diagnostic devices; these do not go through a feature selection that can improve the efficiency of the identification models avoiding redundancy and noise of the processed information [38]. That is why, given the large amount of data generated in the previous subsection, a feature extraction for each variable (acceleration, braking, and steering wheel angle) and GA implementation is proposed, based on previous efforts which show that good results can be obtained [39]. In this way, we would have a total of twenty-one features for each data window, to subsequently evaluate its performance as a classifier or identifier based on the GA and ML approach. These features are presented below.

The Mean (M1) for each data window

(1) $$\bar{x}=\frac{1}{n}\sum_{i=1}^{n}X_{i}$$

The Variance (M2) for each data window

(2) $$\sigma^{2}=\frac{\sum_{i=1}^{n}{\left(X_{i}-\bar{X}\right)}^{2}}{N}$$

The Skewness (M3) for each data window

(3) $$\gamma 1=\frac{\frac{1}{2}\sum_{i=1}^{n}{\left(x_{i}-\bar{x}\right)}^{3}}{{\left[\frac{1}{n-1}\sum_{i=1}^{n}{\left(x_{i}-\bar{x}\right)}^{2}\right]}^{\frac{3}{2}}}$$

The Kurtosis (M4) for each data window

(4) $$K=\frac{\sum_{i=1}^{n}{\left(X_{i}-\bar{X}\right)}^{4}}{N_{s^{4}}}-3$$

The Standard Deviation for each data window

(5) $$\sigma =\sqrt{\sigma^{2}}$$

The Max for each data window

(6) $$X_{\left(n\right)}=max\left\{\begin{array}{c} X_{1},\cdots ,X_{n} \end{array}\right\}$$

The Dynamic range for each data window

(7) $$DR=X_{\left(n\right)}-min\left\{\begin{array}{c} X_{1},\cdots ,X_{n} \end{array}\right\}$$

The data windows taken for feature extraction were 2 s; in the equations, $X_{i}$ is the *i*th raw signal value.

### 2.3. Genetic Algorithm Implementation

A natural selection-inspired optimization algorithm is called GA. It is a population-based search algorithm that makes use of the idea of the strongest individuals surviving. By repeatedly applying the genetic operator to the population’s existing individuals, new populations are created. The main components of GA are chromosome representation, selection, crossover, mutation, and fitness function calculation [40].

For this research work, a genetic algorithm was used to solve optimization problems (GALGO) [41] in contemplation of improving the performance in the driver identification since the performance measure is focused on terms of precision, computational cost, and training time of the classification algorithms for this particular proposal. According to the GALGO tool developed for language R, it does not provide normalization methods to correct systematic errors, so the data must be normalized before analysis. For this case, we used the Z-normalization shown in Equation (8).

(8) $$z_{i}=\frac{x_{i}-\bar{x}}{\sigma }$$

**Figure 4:**
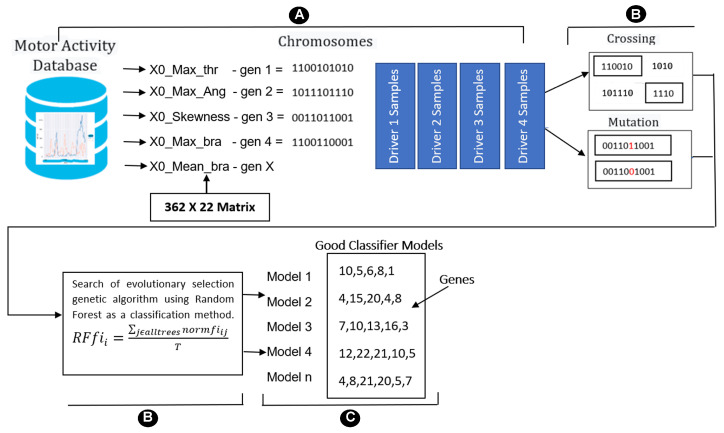
Schematic representation of multivariate variable selection using genetic algorithm.

The general scheme is shown in Figure 4. A genetic algorithm searches for and evolves a combination of genes (chromosomes encoding a multivariate model) that distinguish between classes from a dataset of four classes (A), where $RFfi_{i}$ is the importance of feature *i* calculated from all trees in the random forest model, $normfi_{i}j$ is the normalized feature importance for *i* in tree *j*, and *T* is the total number of trees (B). This process is repeated multiple times to produce a set of models (C). Despite possible differences in gene composition, both models are equally accurate in classifying data. Multiple instances of a gene in various models suggest that the gene is significant for the classification issue in a multivariate setting [41].

The procedure for the implementation of GA was to take the entire dataset obtained previously, to subsequently configure an object called BigBang that will store 1000 chromosomes containing five genes corresponding to develop models using random forest where fitness was evaluated as the accuracy of the model following 3-fold cross-validation.

RFC is a combination of tree predictors such that each tree depends on the values of a random vector sampled independently and with the same distributions for all trees in the forest. The generalization error for forests converges to a limit as the number of trees in the forest becomes large [42]. In general, the process of RF follows the algorithm described below. One leaf is added to the tree’s construction at each stage. To increase forecast accuracy, the decision trees are combined into a single tree at the end [43].

After the genetic search was completed, a Forward Selection (FS) process was performed using all datasets with three cross-validations. The FS method built models by adding features one at a time, evaluating each model’s performance as it went from highest to lowest rank. In this subset of models, the model’s features with the best accuracy are kept, while the others are dropped [18].

In order to extend the experimentation, a data analysis phase was also added for the classification of the different combinations between two drivers, where different feature selection techniques were implemented, such as Recursive Feature Selection (RFE) and least absolute shrinkage and selection operator (LASSO).

### 2.4. Least Absolute Shrinkage and Selection Operator

The feature selector least absolute shrinkage and selection operator (LASSO) is used in this work; the other selectors are smoothed clipped absolute deviation (SCAD), minimax concave penalized likelihood (MCP), stepwise logistic regression, and iterative sure independence screening (ISIS). LASSO is implemented, as a fast and solid solution, from 22 features presented in the driver datasets after preprocessing, and was set and adjusted to a generalized linear model and similar through the penalized maximum likelihood.

The developed LASSO implementation solves the problem:

(9) $$\begin{array}{c} min\\ \left(\beta_{0},\beta \right)\in R^{p+1} \end{array}\left[\frac{1}{2N}\sum_{i=1}^{N}{\left(y_{i}-\beta_{0}-x_{i}^{T}\beta \right)}^{2}+\lambda P_{a}\left(\beta \right)\right],$$

where

(10) $$P_{a}\left(\beta \right)=\left(1-\alpha \right)\frac{1}{2}{∥\beta ∥}_{\ell_{2}}^{2}+\alpha {∥\beta ∥}_{\ell_{1}}$$

(11) $$=\sum_{j=1}^{p}\left[\frac{1}{2}\left(1-\alpha \right)\beta_{j}^{2}+\alpha \left|\beta_{j}\right|\right]$$

this is the elastic-net penalty, as demonstrated by Zou and Hastie (2005) [44]. $P_{\alpha }$ represents a trade-off between the ridge-regression penalty (α=0) and the lasso penalty (α=1). The constant coefficient is $\beta_{0}$, and the vector of coefficients is β. The elastic net penalty is determined by α and serves to bridge the gap between the LASSO regression (α = 1, the default value) and the Ridge regression (α = 0). The tuning parameter λ determines the overall severity of the penalty. This implementation uses a cross-validation approach, which performed a k-fold cross-validation for glmnet and returned a value for λ [45].

### 2.5. Recursive Feature Elimination

Given an external estimator that assigns weights to features, the goal of RFE is to select features by recursively considering smaller and smaller sets of features [46]. In the case of this research work, a combination of RF and RFE was used, the process of which is shown below.

First, the training samples $X_{0}={\left[x_{1},x_{2},\cdots x_{n}\right]}^{T}$, and the labels of different types $y={\left[y_{1},y_{2},\cdots ,y_{n}\right]}^{T}$ are the inputs. Second, a subset of features S=[1,2,...,m] and list of feature ordering R=[] is initialized. Third, we repeat the following steps until S=[]. The training samples are described as $X=X_{0}\left[:,S\right]$. The next steps involve: training the classifier and calculating the importance of the features in the subset *S*; determining the least importance *f*; updating the list of feature ordering R=[S(f),R]; and excluding the feature with minimum criterion S=S−S(f). Finally, the list of feature ordering *R* is the output [47].

### 2.6. Validation

Once the intelligent feature selection phase is finished, a validation process continues under different conditions (classification of two different drivers and four-driver classification), where the most representative features of the dataset are evaluated using different validation metrics such as accuracy, which is calculated as the ratio of true results (true positives and true negatives) between the total number of the observations examined [30], as shown in Equation (9).

(12) $$Accuracy=\frac{tp+tn}{tp+fp+tn+fn}$$

where tp are the true positives, tn are the true negatives, fp are the false positives, and fn are false negatives.

Another validation metric considered is precision, which measures the proportion of true results, among the total number of cases examined: it is a ratio between the correctly predicted observation and the total number of observations. Equation (10) shows how it is calculated.

(13) $$Precision=\frac{tp}{tp+fp}$$

There is also recall, which calculates the proportion of observations that were assigned to a given class (retrieved records), among the observations that actually belong to the class, as shown in Equation (11).

(14) $$Recall=\frac{tp}{tp+fn}$$

Since precision and recall are needed to evaluate the classification capabilities of an algorithm, it is convenient to find a single measure that considers both. This measure is known as F1-Score, which is the harmonic measure of precision and recall. This value is obtained with Equation (12).

(15) $$F_{1}=\frac{precision\cdot recall}{precision+recall}$$

Finally, there are also visual ways to know the performance of an algorithm such as confusion matrices. A confusion matrix is used in machine learning to evaluate and in this case to visualize the behavior of classification models. It is basically a square matrix in which the rows represent the actual classes and columns represent the predicted class [48].

For the experiments, “python 3.7.10” was used in the context of jupyter notebook, a web application for creating and sharing computational documents [49], and R-studio, a software dedicated to sustainable investment in free and open source software for Data Science [50].

## 3. Results

**Figure 5:**
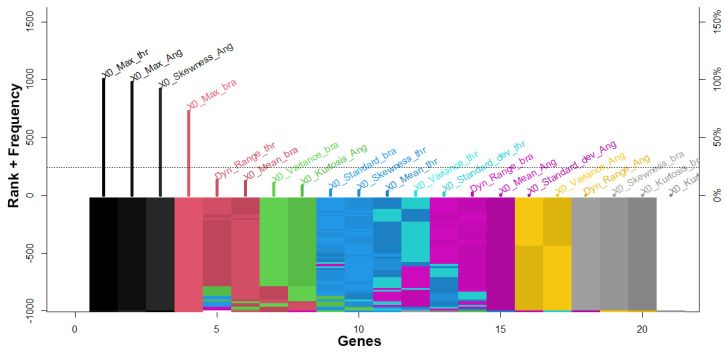
The plot shows the ranks of all genes (horizontal axis) and their frequency in log scale (in the vertical axis) to highlight small frequencies and compact high frequencies.

From the data acquisition, it was mentioned that a total of 9000 observations or samples were obtained for each driver who executed the task of driving in a simulated environment for 3 min obtaining three independent variables such as the movement applied to the steering wheel or steering wheel angle value, the pressure exerted on the brake pedal, and pressure exerted on the acceleration pedal. If the total of samples obtained by each driver were added up, we would have 36,000 observations, their respective three features or variables, and their output value depending on the driver to whom these driving values belong, concerning his behavior in the use of the car. From this first dataset, the set statistical features were extracted [51] for each variable mentioned above based on a 2-second data window, resulting in a total of 356 recordings, with 21 features and four labels for each vehicle driver, yielding 365 × 22 matrix. Using this second dataset, the genetic search generated 1000 models. Figure 5 presents the rank gene stability of the most representative relevant genes obtained through genetic algorithms. It can be observed that the variables max value of acceleration, and max and skewness steering wheel angle have better performance to identify four drivers. Taking into account that the number of features obtained through the GA is much lower compared to the number of features proposed by other works, we can say that the computational time and cost are much lower due to the low number of information mathematically selected to process the data.

**Figure 6:**
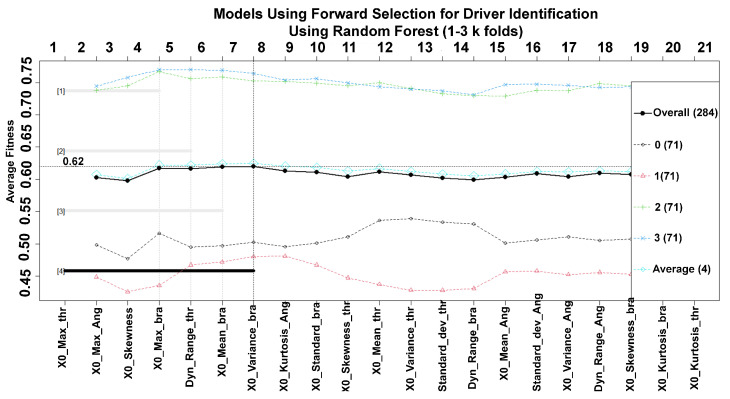
Forward selection method. The solid line represents average accuracy. Colored lines represent the accuracy per class. The best model is 4, which is formed from the first gene up to the seventh gene.

Nevertheless, it is necessary to know the accuracy rate of these variables to distinguish one driver from another. Classic forward selection, which adds one gene at a time, starting with the most and least common, is an easy strategy for this test. Figure 6 shows the results obtained from the method, where there is an overall accuracy of 0.62, which in percentage terms is equivalent to 62% for identifying four drivers. This graph shows some important aspects about the low level of accuracy since the value exposes a general value, which means an average accuracy of all classes. In the case of drivers with identifiers 0 and 1 presenting very low indices, this could mean that behaviors while driving could be very similar between both drivers, which would make it difficult for the classification models to discriminate between one subject and another to have good performance.

To expand the results obtained, an experiment was generated where only two drivers were identified, but based on the most representative variables obtained in the process of GA, Table 2 shows the results obtained for each combination of the four drivers who performed the tests, where 70% of the samples were used for model training and remaining 30% as a test. The classification tests were conducted with the RFC machine learning algorithm, whereas with Grid Search with Cross Validation (GridSearchCV), an exhaustive search of certain candidate parameters was carried out, through loops and testing various possibilities. The best performing parameters were the maximum depth of 15, maximum features of 1, minimum samples per leaf of 15, minimum split samples of 10, and 5 estimators.

**Figure 7:**
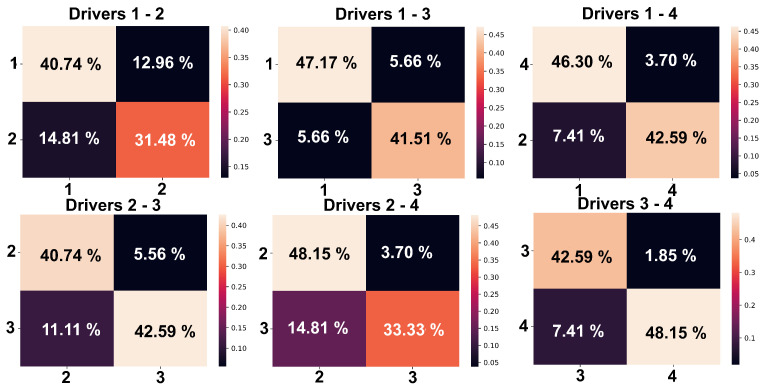
Percentage confusion matrices obtained in the classification of two drivers using 3 statistical variables selected by GA.

The values presented above make sense since drivers 1 and 2 are the ones with the lowest performance, which caused the accuracy when generating classification models using forward selection to decrease. To have a visual overview of these results, Figure 7 shows the confusion matrices obtained from the classification models between a driver combination of the four study subjects used.

With these matrices, it can be demonstrated that the classifications rate of the model for two subjects using intelligent statistical feature selection and RFC can be improved, although the results cannot be compared with those of other authors using data obtained in different conditions (real environments) and that can even discriminate several study subjects with the same classification model, the bias that can be caused by the ever-changing conditions in which the experimentation is carried out will always be present.

The results presented show that, in the case of identifying two drivers, the implementation of GA turns out to be a very efficient technique for the optimization of classification problems since by decreasing the number of features, the information is significantly diminished, which leads it to generate faster models to train with a high percentage of accuracy. It should be noted that, having already a fixed base of features resulting from GA, carrying out a retraining process with different data would be much faster and easier.

On the other hand, there are different feature selection techniques such as Principal Component Analysis (PCA) which are often used to find feature patterns associated with a certain behavior. Priyadharshini et al. [52] conducted research work to identify drivers through data extracted from the On Board Diagnostic II sensor. The main objective of the work is to extract the most important features instead of entering all the features in the machine learning algorithms using PCA, obtaining an accuracy of 75% reducing times, and increasing accuracy. Typically, the first two or three PCA components are used to determine whether individuals can be grouped into two classifications groups based on given criteria. However, there may be a combination of other components that better distinguish individuals. The GA can be useful and efficient to find the best combination of variables for the construction of a classification model [53].

To make a more robust approach, another two feature selection methods and a Random Forest model were implemented as complements. The results in LASSO were in ranges from 0.6 to 1 in AUC, as the partitions were aleatory in the 70% train–30% test (the same partition that is implemented by default in our approach). Another condition sustained in the selection of the model resulting from LASSO was that the model had to have four features; this worked with almost every set except with the 3 and 4 drivers set which had six features as a minimum in all runs concreted (see Table 3).

The other method was RFE, a GA algorithm implemented in python. The results in RFE were in ranges from 0.5 to 1 in AUC, as the partitions were aleatory in the 70% train–30% test (the same partition that is implemented by default in our approach). Another condition sustained in the selection of the model resulting from RFE was that the model had to have four features, and this worked with every set (see Table 4).

## 4. Discussion

Continuing with the discussion of these results, it could be stated that, compared to other research, being able to obtain a system capable of identifying a greater number of vehicle users would be a very complicated task. The conditions in which driving is carried out are not the same; for example, many researchers focus their efforts on the analysis and processing of data sets obtained in real driving environments such as the security dataset [54], the HcilLab [55], and the data set of real-world driving to assess driver workload [56]. However, in the case of work carried out in simulated environments, although also under different circumstances, the results are not very promising either. They confirm that the control of the environment can be considered as an important part of data collection to obtain more objective and precise systems.

Another approach to take into account for driver identification follows Mainardi et al. [57], who computed the average number of drivers per vehicle for the UK region since this value strongly depends on the geosocial conditions. From the publicly available government data, the average number of adults who drive a car is 1.095 per vehicle. Another study says that, in 1997, the US average vehicle occupancy was 1.87 persons per vehicle and in 2019, the average car occupancy was 1.5 persons per vehicle in 2017. Twenty-four percent of US households had three or more vehicles [58]. It could be said that it is not necessary to identify numerous drivers of a single vehicle, given the statistics presented by the previous study, and that can be reflected in other countries.

Some recent papers implemented simulation as a method for data acquisition. Table 5 shows details of the results obtained by other researchers for driver identification using machine learning techniques and driving simulators; it also includes a real driving test [59] so it can be compared as well. Our approach contains features similar to all approaches compared in simulation experiments. The real driving experiment was taken into account so we can compare the driver behavior beyond the driver activity that is not strictly related to the vehicle conduction (image processing) and make clear that it is not strictly needed to identify a driver, reducing substantially the computational cost.

According to the table presented, several interesting aspects can be discussed. This is because the three approaches in state-of-the-art present very important results, in addition to contributing to the automotive field. Yang et al. [60] include many more features, which would imply more processing time added to the driving time to achieve data collection; on the other hand, it must be remembered that, in the proposed methodology, the interval of time shown in the table is the range used for feature extraction. The real time needed for identification is 3 min of active driving, which a very important point that every work should mention; even so, the performance of this job has a significant improvement with only three features. In the case of Heidecker et al. [35], they consider a smaller number of features, and this lasts longer but has much higher performance. However, it uses an Encephalogram (EEC) for data acquisition, which implies an invasive method that could even hinder driving and the user’s experience. Li et al. [61] provide a simulation driving experiment with sensors and provide a very close approach to ours by using four features and a random forest as one of the models implemented; in this work they sustain that the random forest model provides the best performance results. Finally, we analyze the proposal of Xing et al. [59] to make a performance comparison of our approach in a simulated environment against a real driving experiment using image processing; even with all sensors and cameras included in the experiment, the performance was near our approach.

Based on the results presented, it was shown that a feature selection phase in the methodologies that have been proposed can improve the redeeming of machine learning models for driver identification. This is thanks to the optimization of power and processing time.

## 5. Conclusions

In conclusion, it can be said that experimental works in simulated environments and objective intelligent selection of statistical features can improve driver identification models. The proposed methodology shows that these factors are considered necessary for making driver identification models have a performance of 90.74% to identify two drivers and 62 % to identify four in a correct way using a novelty intelligent feature selection with GA. This process allows determining which variables are most relevant for a classification task, in order to increase the levels of performance, precision, and confidence of the algorithms, significantly improving training times and costs.

Some existing methods are based on specific scenarios and, even more importantly, in vehicles of a particular brand, a problem that can be solved by creating more general systems using a simulator that is closer to the experience of actual driving. Once the experimentation phase is finished, only a slight adjustment of configurations would be necessary for its implementation in any brand of car.

It is a fact that, nowadays, it is more and more necessary to use one’s vehicle due to different circumstances. This means that, as the statistics presented above show, the maximum number of drivers per vehicle could be two, but this is not conclusive data, which is why it is proposed in future work to increase the active driving time as well as the data windows for feature extraction, in order to continue with the same scheme of intelligent selection of variables for improvement and generation of an optimal, efficient, objective, and, above all, reliable driver identification system.

## Figures and Tables

**Table 1:** Example of generating data by each driver from sensors of the CARLA simulator.

Sample Number	Motor Activity Acceleration	Motor Activity Brake	Motor Activity SWA	Driver
1	0.679932302	0	−0.243051659	0
2	0.672789414	0	0.301507503	0
3	0.669198607	0	−0.104604312	0
…	…	…	…	…
1	0	0.5495534057	−1.439965432	1
2	0	0.392749183	−0.079991518	1
3	0.524402881	0	−0.590714625	1
…	…	…	…	…
1	0	0.898714153	2.221737421	2
2	0.476545161	0	−1.892375448	2
3	0.402442758	0	3.758628905	2
…	…	…	…	…
1	0.840223762	0	8.276900048	3
2	0.639982157	0	−16.88071172	3
2	0.639982157	0	−16.88071172	3

**Table 2:** Validation Results With AG Features Selection for Two Driver Identification using RFC.

Driver	Validation Metrics		
	Precision	Recall	F1-Score
1	0.69	0.83	0.71
2	0.82	0.83	0.71
**Accuracy**			**72.2%**
1	0.89	0.89	0.89
3	0.88	0.88	0.88
**Accuracy**			**88.68%**
1	0.86	0.93	0.89
4	0.92	0.85	0.88
**Accuracy**			**88.89%**
2	0.79	0.88	0.83
3	0.88	0.79	0.84
**Accuracy**			**83.33%**
2	0.76	0.93	0.84
4	0.90	0.69	0.78
**Accuracy**			**81.48%**
3	0.85	0.96	0.90
4	0.96	0.87	0.91
**Accuracy**			**90.74%**

**Table 3:** LASSO selection with Random Forest model implementation metrics.

Driver Set	Features	AUC	Accuracy	Precision	Recall	$F_{1}$ Score
1-2	“X0_Mean_Ang”, “X0_Max_bra”, “X0_Mean_thr”, “X0_Max_thr”	0.817 (0.702–0.931)	0.9423	0.9231	0.9231	0.9412
1-3	“X0_Mean_Ang”, “X0_Kurtosis_Ang”, “X0_Kurtosis_bra”, “X0_Max_thr”	0.885 (0.790–0.979)	0.9615	0.9615	0.9615	0.9615
1-4	“X0_Mean_bra”, “X0_Skewness_bra”, “X0_Standard_bra”, “Dyn_Range_thr”	0.816 (0.692–0.940)	0.7115	0.9615	0.9615	0.7692
2-3	“X0_Mean_Ang”, “X0_Standard_dev_Ang”, “X0_Mean_thr”, “X0_Max_thr”	0.925 (0.858–0.991)	0.9423	0.9231	0.9231	0.9412
2-4	“X0_Mean_Ang”, “X0_Kurtosis_Ang”, “X0_Mean_bra”, “X0_Variance_thr”	0.815 (0.699–0.932)	0.8846	1	1	0.8966
3-4	“X0_Mean_Ang”, “X0_Skewness_Ang”, “X0_Kurtosis_Ang”, “X0_Mean_bra”, “X0_Mean_thr”, “X0_Max_thr”	0.949 (0.877–1)	0.9231	1	1	0.9286

**Table 4:** RFE selection with Random Forest model implementation metrics.

Driver Set	Features	AUC	Accuracy	Precision	Recall	$F_{1}$ Score
1-2	“X0_Mean_Ang”, “X0_Max_Ang”, “X0_Mean_thr”, “X0_Max_thr”	0.71 (0.566–0.854)	0.8269	0.8462	0.8462	0.8302
1-3	“X0_Variance_Ang”, “X0_Mean_thr”, “X0_Max_thr”, “Dyn_Range_thr”	0.902 (0.825–0.98)	0.9231	0.8462	0.8462	0.9167
1-4	“X0_Standard_dev_Ang”, “X0_Max_Ang”, “X0_Mean_thr”, “X0_Max_thr”	0.904 (0.827–0.981)	0.9615	0.9615	0.9615	0.9615
2-3	“Dyn_Range_Ang”, “X0_Mean_thr”, “X0_Max_thr”, “Dyn_Range_thr”	0.922 (0.837–1)	0.9231	0.8462	0.8462	0.9167
2-4	“X0_Mean_Ang”, “X0_Max_Ang”, “X0_Mean_thr”, “X0_Max_thr”	0.831 (0.718–0.945)	0.9038	0.9615	0.9615	0.9091
3-4	“Dyn_Range_Ang”, “X0_Max_bra”, “X0_Mean_thr”, “X0_Max_thr”	0.962 (0.894–1)	0.9615	1	1	0.963

**Table 5:** Results obtained from proposed work from past investigations that also use simulations environments.

Title	Technique	Validation Metric	Result	Features	Time
Driver2vec: Driver Identification from Automotive Data [60] (2020)	temporal convolutional networks, embedding separation power of triplet loss and classification accuracy of gradient boosting decision trees	Accuracy	83.1%	31	10 s
Novelty Based Driver Identification on RR Intervals from ECG Data [35] (2021)	Combined Approach to Novelty Detection in Intelligent Embedded Systems (CANDIES), Gaussian Mixture Model (GMM), one-class SVM classification	precision, recall, $f_{1}$ score	Unknown, driver 1: 56.8%, 76.6%, 59.8%; Unknown, driver 1/2: 43.5%, 64%, 47.7%; Unknown, driver 1/2/3: 37.83%, 56.12%, 42.38%	9	1 min
Driver identification in intelligent vehicle systems using machine learning algorithms [61] (2018)	K-nearest neighbor (KNN) algorithm, random forests (RFs) algorithm, multilayer perceptron algorithm (MLP), Adaboost algorithm, ensemble	accuracy, recall, precision	Best performance model Random Forest: 93.7%, 93.7%, 93.4%	4	100 records per sec
Driver Activity Recognition for Intelligent Vehicles: A Deep Learning Approach [59] (2019)	deep convolutional neural networks (CNN), Gaussian mixture model	accuracy	81.6% accuracy using the AlexNet, 78.6% and 74.9% accuracy using the GoogLeNet and ResNet50	pre-trained sets	-
**This Work in** **1-2 drivers dataset**	**Genetic Algorithm** **with GALGO-rf**	**Accuracy**	**72.2%**	4	2 s
**This Work in** **1-3 drivers dataset**	**Genetic Algorithm** **with GALGO-rf**	**Accuracy**	**88.68%**	4	2 s
**This Work in** **1-4 drivers dataset**	**Genetic Algorithm** **with GALGO-rf**	**Accuracy**	**88.89%**	4	2 s
**This Work in** **2-3 drivers dataset**	**Genetic Algorithm** **with GALGO-rf**	**Accuracy**	**83.33%**	4	2 s
**This Work in** **2-4 drivers dataset**	**Genetic Algorithm** **with GALGO-rf**	**Accuracy**	**81.48%**	4	2 s
**This Work in** **3-4 drivers dataset**	**Genetic Algorithm** **with GALGO-rf**	**Accuracy**	**90.74%**	3	2 s

Bold text represents these work models with the highest Average accuracy in each drivers dataset.

## Data Availability

The dataset can be accessed via: https://github.com/Dantecore2/DriverID_Dateset, accessed on 26 October 2022. It contains the following: one file .csv with statistical data of steering wheel angle and pressure of brake and accelerations pedals. The .rar file has all the data acquisition obtained through experimental tests.
